# High-output chyloperitoneum following laparoscopic Nissen fundoplication treated with retrograde transvenous thoracic duct embolization

**DOI:** 10.1186/s42155-020-00110-9

**Published:** 2020-04-06

**Authors:** Gernot Rott, Frieder Boecker

**Affiliations:** Department of Radiology, Bethesda-Hospital Duisburg, Heerstr. 219, 47053 Duisburg, Germany

**Keywords:** Chyloperitoneum, Chylous ascites, Nissen fundoplication, Thoracic duct embolization, Retrograde transvenous access

## Abstract

**Background:**

Iatrogenic injury of the thoracic duct with clinical significant chyloperitoneum is a rare complication of abdominal surgery. Chyloperitoneum following laparoscopic Nissen fundoplication has been described in a few cases only. Most interventionists use the antegrade transperitoneal approach for thoracic duct embolization.

**Case presentation:**

A 61-year-old woman had been operated with laparoscopic Nissen fundoplication and hiatoplasty. A few weeks later she presented with high-output chyloperitoneum due to large leakage of the proximal thoracic duct. Conservative treatment and conventional transnodal lymphangiography did not result in a significant improvement. Thoracic duct embolization via retrograde transvenous access was challenging but both technically and clinically successful.

**Conclusion:**

To the best of our knowledge, this is the first case-report about thoracic duct embolization with retrograde transvenous access in the rare situation of chylous ascites following laparoscopic fundoplication. Thoracic duct embolization with the seldom used retrograde transvenous access may be the more physiologic and safer route in doing this and might be used as treatment of first choice.

## Background

Iatrogenic traumas of the thoracic duct (TD) with clinically significant lymph leakages are uncommon but well-known complications of thoracic surgery. They may present as chylothorax, in particular after esophagectomy and very rarely occur after abdominal surgery presenting as chyloperitoneum (Lv et al. 2017; Chen and Itkin 2011). Chylothorax and chyloperitoneum are categorised as low-output (< 500 mL/day) or high-output (> 500 mL/day) based on the output documented by the pleural or peritoneal drain (Delaney et al. 2017). Low-output chyle leaks may be effectively treated with conservative management, whereas high-output leakages often require additional therapy. Currently, thoracic duct embolization (TDE) is considered to be the gold standard for treatment. Here, by far the most interventionists primarily use the antegrade transperitoneal access.

## Case presentation

**Fig. 1:**
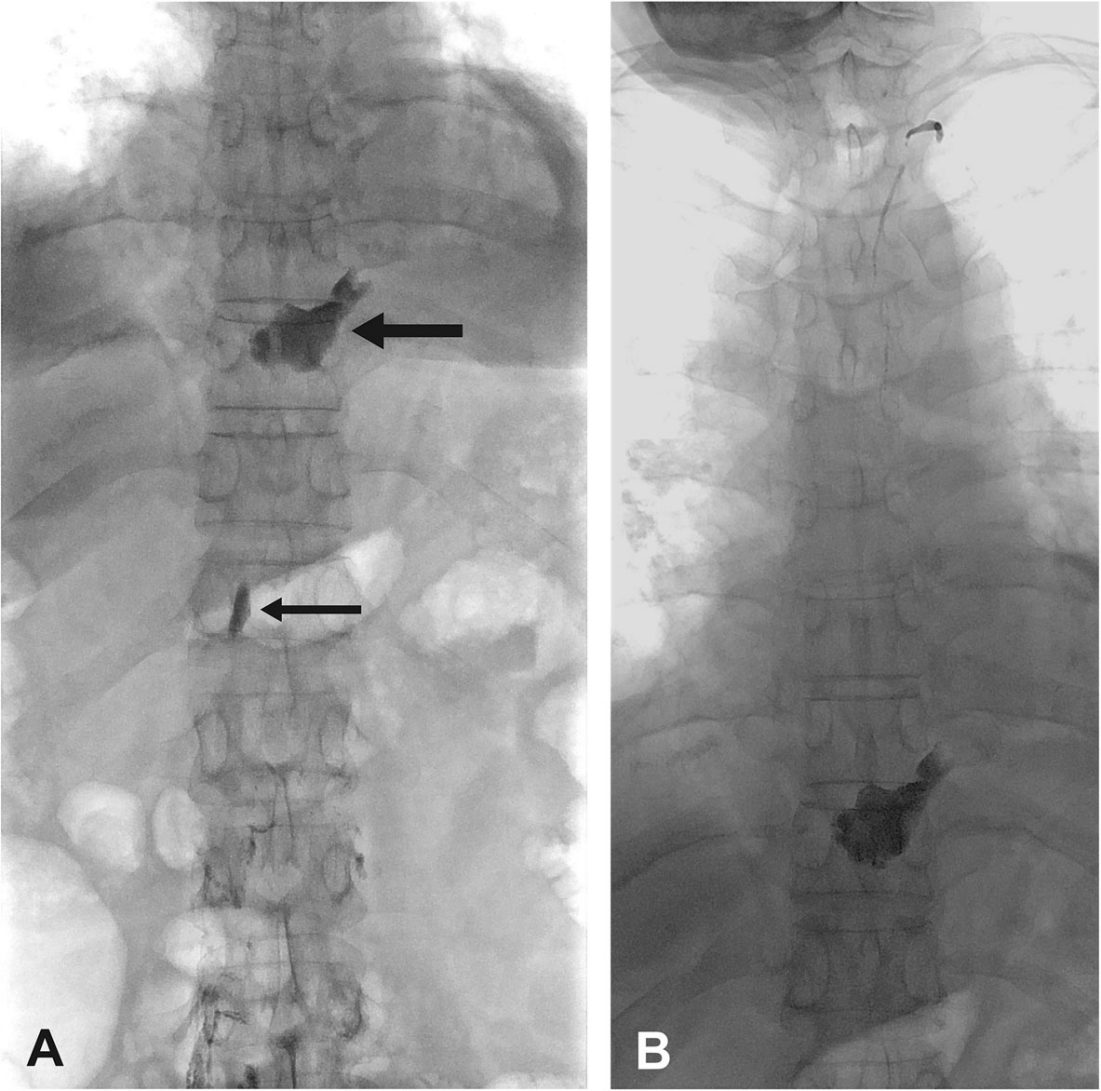
Transnodal lymphangiography. **a** Cisterna chyli at the T12 vertebral level (small black arrow) and a lymph leakage of the thoracic duct at the T10 vertebral level in the posterior inferior mediastinum (large black arrow). **b** Leakage of the thoracic duct at the T10 vertebral level and typical course of the cervical part of the thoracic duct, probably with the „simple type“

**Fig. 2:**
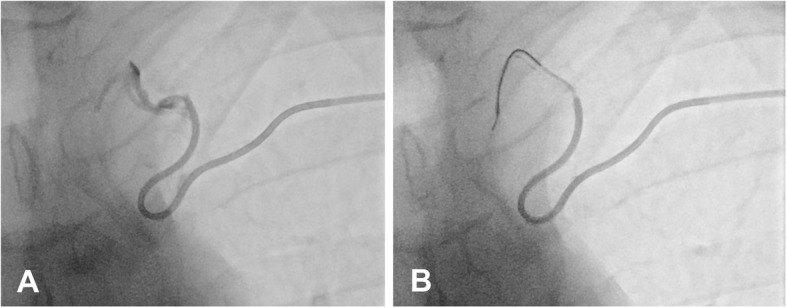
Retrograde transvenous embolization of the thoracic duct. **a** Mikaelsson catheter in the left subclavian vein with ductography of the cervical course of the thoracic duct. **b** Microcatheter inserted in the cervical part of thoracic duct

A 61-year-old woman presented 2 months after laparoscopic Nissen fundoplication and hiatoplasty with persistent chylous ascites. Computed tomography showed ascites in the lower posterior mediastinum and upper abdomen with hiatal insufficiency. Conservative treatment including continuous peritoneal drainage with up to 700 mL/day drained fluid proved unsuccessful. Conventional transnodal lymphangiography (Fig. 1a, b) revealed the expected large lymph leakage of the proximal TD at the T10 vertebral level and very sparsely filling of the TD with the cervical part seeming to be of the so-called simple type (Kariya et al. 2018). As expected, lymphangiography induced only a temporary volume drop of chyle output. A few days later, TDE with retrograde transvenous access was performed:

A 5F-sheath (Radifocus Introducer II, Terumo, Tokyo, Japan) was introduced via a cubital brachial vein, the ostial valve of the TD was intubated with a 5F-Mikaelsson-catheter (Imager II, Boston Scientific, Marlborough, USA) and a 2.7F-microcatheter (Progreat, Terumo) was inserted into the TD (Fig. 2a, b). The cannulation of the TD then was performed both with the corresponding 0.021-in. microguidewire and a 0.012-in. microguidewire (Radifocus Guidewire GT, Terumo).

**Fig. 3:**
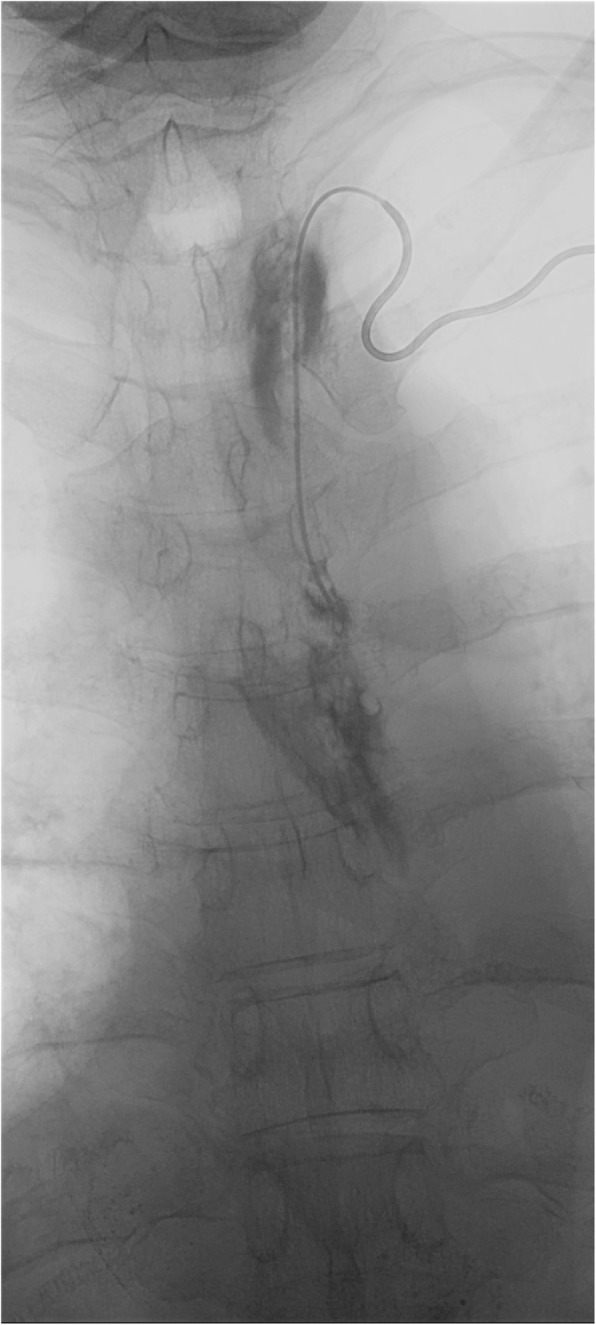
Retrograde transvenous embolization of the thoracic duct. Microcatheter in the distal thoracic duct with some contrast medium extravasation in the upper mediastinum due to slight damages of the thoracic duct with the microguidewire

**Fig. 4:**
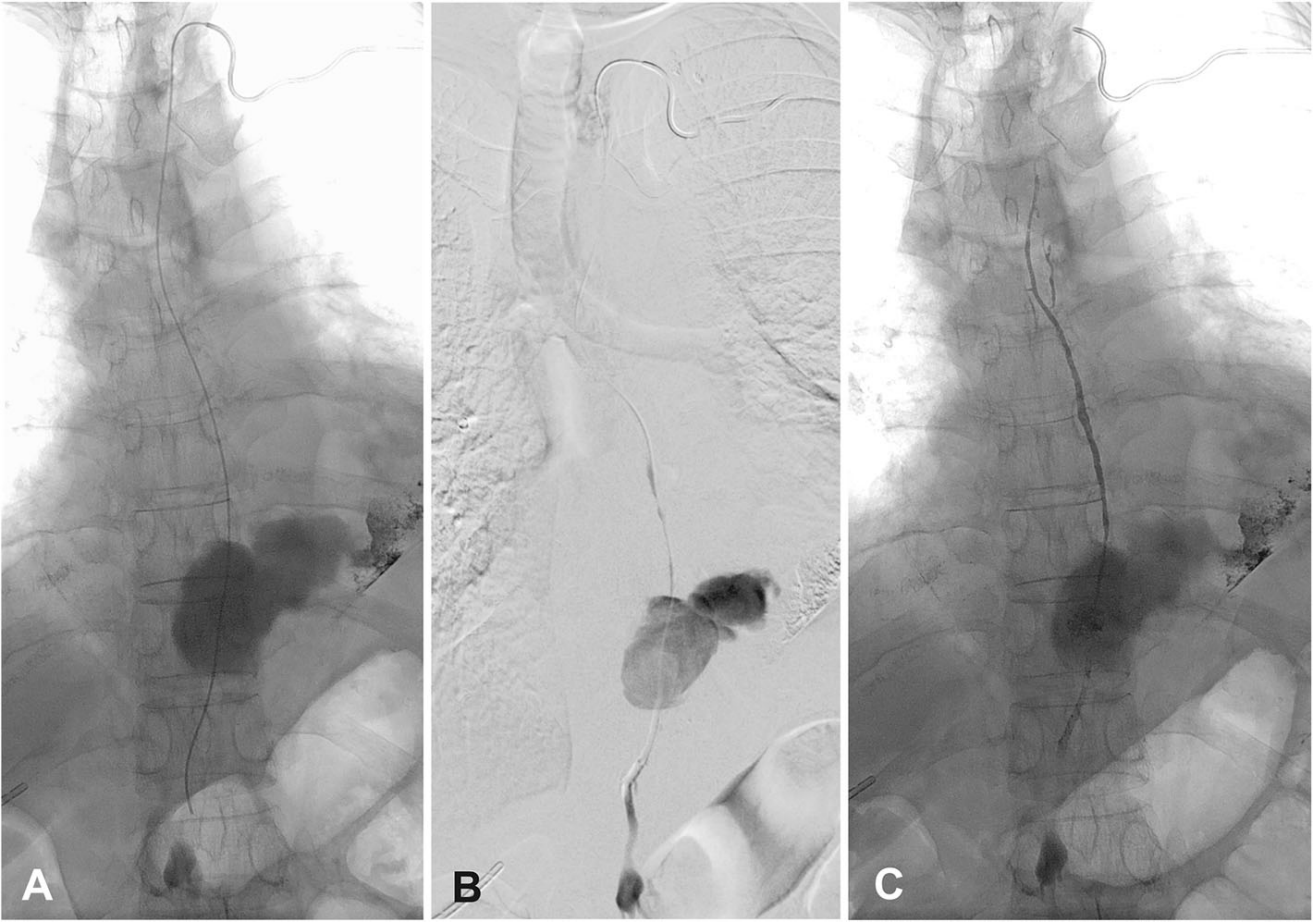
Retrograde transvenous embolization of the thoracic duct. **a** Microcatheter in the proximal thoracic duct direct above the cisterna chyli with large chylous leakage at the T10 vertebral level including residual lipiodol-leakage from the transnodal lymphangiography performed few days before. **b** Same situation documented with digital-subtraction-angiography. **c** Situation after thoracic duct embolization with glue cast from direct above the cisterna chyli up to the distal thoracic duct

Due to an overall small calibre of the TD further advancing of the microcatheter was challenging and time-consuming. In doing so slight damages of the thoracic part of the TD with contrast medium extravasation into the upper mediastinum due to microcatheter-manipulations occurred (Fig. 3). Eventually, the microcatheter could be placed beyond the leakage site and into the cisterna chyli. Lymphangiography showed the large leakage at the T10 vertebral level filling parts of the lower posterior mediastinum (Fig. 4a, b). Thereafter, a 1,5 mL mixture of n-butyl-2-cyanoacrylate and iodized oil (NBCA:Lipiodol = 1:3) was injected into the TD beginning directly above the cisterna chyli at the T11 vertebral level, over the leakage site at the T9–10 vertebral level and ending at the T5 vertebral level while slowly withdrawing the microcatheter (Fig. 4c).

Promptly after embolization chyle output stopped. Post-interventional course was uneventful. To be on the safe side the peritoneal drain was left in place for 5 days, and the patient was discharged the sixth day after embolization without further measures. A telephone call 3 weeks after TDE did not reveal any evidence of chylous leak recurrence or complication of the procedure.

## Discussion

Thoracic duct embolization used as a generic term includes different types of image-guided accesses to the TD: The frequently used percutaneous antegrade access, usually transperitoneal and quite rarely also retroperitoneal (Pamarthi et al. 2014; Itkin et al. 2010; Nadolski and Itkin 2013), and the less frequently used retrograde access, usually transvenous, rarely directly transcutaneous (Pamarthi et al. 2014; Mittleider et al. 2008; Koike et al. 2013).

The transperitoneal antegrade access carries the risk of peritoneal organ penetration including arteries, the biliary system and the intestine with an estimated morbidity rate of about 10% (Pieper 2018). Venous, lymphatic, biliary and other complications, including pulmonary emboli, pancreatitis and peritonitis have been described (Schild and Pieper 2020) and are related to the transabdominal route of the needle and microcatheter.

The transvenous retrograde access may technically be considered more challenging, as it requires retrograde intubation of the lymphovenous junction with the ostial valve as well as the insertion of a microcatheter possibly the long way down of the whole TD including all retrograde valve-passages (Koike et al. 2013). In cases of complete transection of the TD, a retrograde approach may not be expedient because it might be impossible to cross the lesion and enter the proximal section of the TD (Pieper and Schild 2015). Apart from that it avoids possible injuries of intraperitoneal organs and structures and therefore is the substantially less invasive and more physiologic approach to the TD. In addition, the retrograde transvenous access is associated with much less discomfort for the patient and so, unlike the transabdominal access, does not require analgosedation or general anaesthesia.

Retrograde transvenous TDE was first described by Mittleider et al. in 2008 (Mittleider et al. 2008). In contrast to antegrade trans- or retroperitoneal TDE, there are only very few case reports on retrograde transvenous TDE (Kariya et al. 2018; Mittleider et al. 2008; Koike et al. 2013; Chung et al. 2015). In the meta-analysis of Kim et al. (Kim et al. 2018) of 9 publications with 310 cases of TDE technical success was achieved in 62.9%. However, only 2 of 310 (0,6%) TDEs have been performed with a retrograde transvenous approach. Kim et al. come to the conclusion, that TDE is associated with high clinical success and low technical success. The recent publication of Kariya et al. about „transvenous retrograde thoracic ductography“, which is technically comparable to retrograde transvenous TDE, reports a technical success rate of 61.5% in 13 cases with significantly higher rates in patients with the so-called simple type (80%) than with the so-called plexiform type of the cervical part of the TD. However, the catheter could be inserted to the cisterna chyli in only 46.2% of patients. Kariya states „this technique is safe and does not require any special devices or instruments “(Kariya et al. 2018).

In the case we present, catheterisation of the TD was quite challenging mainly due to a very small calibre of the TD. The probable reason for this was poor filling of the distal TD due to the large pro-ximal leakage, as expected in a high-output situation. This specific problem of the retrograde approach has been addressed theoretically in a publication by Chung et al. (Chung et al. 2015) and is confirmed by our case. During our intervention minimal injuries of the TD with subsequent contrast medium extravasation due to guidewire-manipulations occurred. We continued the procedure being convinced that such incidents will not cause complications, in particular as long as the TD is embolized afterwards.

A literature search in the databases PubMed and LIVIVO using the terms “laparoscopic fundoplication AND chylous ascites” and “laparoscopic fundoplication AND chyloperitoneum” was conducted. We found 8 publications with 9 corresponding cases. Only one of these was treated with TDE, however with the usual antegrade transabdominal approach and direct glue embolization via the needle without cannulation of the TD (Hwang et al. 2012). Thus, to the best of our knowledge our case is the first one reported of chylous ascites following fundoplication effectively treated with retrograde transvenous TDE.

## Conclusion

This case report illustrates that retrograde transvenous TDE is feasible even in the setting of TD-injury following surgery with high-output chyloperitoneum, where the TD is exceedingly small because of sparse filling due to a large lymph leakage. As about one third of antegrade TDE-attempts turn out to be technically unfeasible, the less invasive retrograde approach for TDE appears to be a valuable option for TDE and should be taken into account more frequently.

## Data Availability

Not applicable.

## Abbreviations

TD: Thoracic duct
TDE: Thoracic duct embolization
